# Understanding non-partner sexual violence perpetration in young Tanzanian men: a cross-sectional study

**DOI:** 10.1186/s12889-025-23248-4

**Published:** 2025-05-30

**Authors:** Rebecca Brambilla, Gerry Mshana, Neema Mosha, Andrew Gibbs, Donati Malibwa, Saidi Kapiga, Heidi Stöckl

**Affiliations:** 1 Institute for Medical Information Processing, Biometry and Epidemiology (IBE), Faculty of Medicine, LMU Munich, Munich, Germany; 2 Pettenkofer School of Public Health, Munich, Germany; 3 Mwanza Intervention Trials Unit (MITU), Mwanza, Tanzania; 4 Department of Psychology, University of Exeter, Exeter, United Kingdom; 5 Gender and Health Research Unit, South African Medical Research Council, Cape Town, South Africa; 6 Department of Global Health and Development, London School of Hygiene and Tropical Medicine (LSHTM), London, United Kingdom

**Keywords:** Non-partner sexual violence, Non-partner rape, Sexual harassment, Perpetration, Young men, Masculinities

## Abstract

**Background:**

The World Health Organisation estimates that worldwide six percent of women aged 15–49 have experienced non-partner sexual violence (NPSV) in their lifetimes. A similar prevalence is found in sub-Saharan Africa. This form of violence is comparatively under-researched, leading to a dearth of knowledge around potential risk factors for male perpetration of NPSV.

**Methods:**

We sought to explore key risk factors for perpetration of non-partner rape, verbal and physical sexual harassment in young Tanzanian men by conducting a cross-sectional survey of 1002 young men aged 18 to 24 living in Mwanza, Tanzania between June 2021 and March 2022. We conducted unadjusted logistic regression for bivariate associations between all three forms of NPSV and risk factors at the sociodemographic, behavioural, and mental health and substance misuse level. The risk factors independently associated with the outcomes were included in three separate multivariable logistic regression models. We then used dominance analysis to determine which factors had the strongest association with all three forms of NPSV perpetration.

**Results:**

Among the young men in our sample, 9% reported having perpetrated non-partner rape (*n* = 86), 19% having physically harassed a woman (*n* = 188) and 33% having verbally harassed a woman (*n* = 330). After adjustment for the other risk factors in the model, pornography consumption, having multiple sexual partners, gambling, and depressive symptoms remained significantly associated with more than one form of NPSV.

**Conclusions:**

The widespread nature of sexual harassment and rape perpetration among young men in our study and the associated risk factors, which are all tied to notions of masculinity encouraging domination, promiscuity, and risky health behaviours, call for harmful gender norms to be addressed to reduce the incidence of NPSV.

**Supplementary Information:**

The online version contains supplementary material available at 10.1186/s12889-025-23248-4.

## Background

### Definitions and measurement of non-partner sexual violence

Since the Fourth World Conference on Women held in Beijing nearly 30 years ago and the Beijing Declaration and Platform of Action [1], in which governments committed themselves to eradicate violence against women and girls in all its forms, much research has been undertaken on the prevalence, predictors, consequences and the healthcare sector burden of intimate partner violence (IPV), that is violence between people who are (or were) in an intimate relationship. Comparatively less research has been conducted on non-partner sexual violence (NPSV) [2], namely sexual violence perpetrated by a person who was not in an intimate relationship with the victim, whether they were an acquaintance or entirely unknown. The development of a common definition and relevant measurement tools for NPSV has lagged behind that for IPV [3]. As a result, we know much less about the global, national and population prevalence of NPSV, the factors shaping perpetration and the ramifications for people who experience it.

Whereas the most recent estimates of the population-prevalence of IPV against women include data from 366 eligible studies from 161 countries [4], the equivalent for NPSV only encompasses 227 studies from 137 countries [5]. Furthermore, often only a single item related to forced sex/sexual acts is used in surveys to measure NPSV, compared to a number of validated scales using acts-based questions to capture prevalence of IPV [5]. Therefore, the phenomenon of sexual violence by a non-partner remains much less clear, due to discrepancies and inconsistencies in measurement tools, as well as widespread underreporting caused by fear, shame and social stigma [6], leading to a likely significant underestimation of its magnitude and impact.

### Prevalence of non-partner sexual violence

Despite the shortcomings in measurement and the fewer population-prevalence estimates of NPSV, the global lifetime prevalence of non-partner rape against women aged 15–49 years was estimated at 6.0% [5], with substantial variation across regions. Current estimates show sub-Saharan Africa experiencing a burden similar to the worldwide estimates, also at 6.0% [5]. The most recent data for Tanzania found that 3.3% of women aged 15–49 reported having ever experienced sexual violence by a non-intimate partner [7], an estimate which might suffer from significant underreporting, given the low-propensity for reported health-seeking behaviour after sexual violence experience in this population [7].

The phenomenon of sexual harassment, despite increased attention in recent years, remains even more elusive than NPSV. Broadly, the term is used to cover a number of unwelcome behaviours of a sexual nature, both physical and verbal. Given the lack of formal conceptualization and consensus over the meanings and manifestations of sexual harassment, the development of measurement tools is challenging. There are no current worldwide estimates for these behaviours and instances of sexual harassment are not captured in most nationally-representative surveys, with only 23 countries measuring sexual harassment specifically [8]. Similarly, a systematic review on prevalence of sexual harassment in low- and middle-income countries (LMICs) uncovered unclear and inconsistent definitions of sexual harassment and that most studies did not use a validated measurement tool [9].

In qualitative data from Tanzania, the concept of sexual harassment was found to be very fluid, tied to notions of gendered power and consent [10]. What constituted harassment for either men or women was strictly linked to the role of the harasser, whether or not they are in a position of power over the person being harassed, or in charge of material resources, and whether the power relationship allows for true consent to be given. Moreover, verbal sexual harassment was more likely to be accepted if coming from an acquaintance, than from a stranger. How courtship rituals manifested themselves, and whether the sexual advances were wanted or not, seem to be key in understanding what can be classified as “sexual harassment” in this setting.

For the purposes of this paper, we define NPSV as encompassing instances of sexual violence that include “rape, attempted rape or sexual assault” [5] (i.e. non-partner rape) and events of sexual harassment, which cover “repetitive and unwelcome sexual behaviors, encompassing verbal, physical, psychological, and visual forms” [11] towards somebody who is not a current or former partner.

### Risk factors for non-partner sexual violence

The lack of consistent definitions and reliable estimates of NSPV has made it harder not only to measure, but also to understand the risk factors driving this behaviour. Some overlaps have been found between predictors of IPV and NPSV [3], such as unemployment [12, 13] and low educational attainment [14], as well as some behavioural and mental health-related factors [15]. However, given the fact that less evidence of predictors of NPSV perpetration exists and that not all men who perpetrate IPV also perpetrate NPSV and vice-versa, it is crucial to investigate which variables underlie male perpetration of NPSV.

**Fig. 1:**
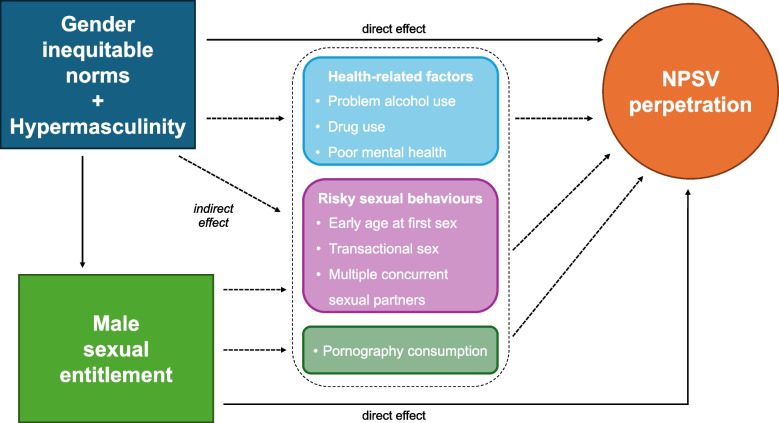
Direct and indirect links between inequitable gender norms and hypermasculinity, male sexual entitlement, and NPSV perpetration

There are some known structural-level drivers of NPSV, namely political conflict [16] and legal systems that hinder prosecution [17]. Yet, findings from the UN Multi-country Study on Men and Violence in Asia and the Pacific highlight that the most common motivation for perpetrating rape reported by men was sexual entitlement [14]. Similarly, a study on factors associated with rape conducted in South Africa concluded that ideas of sexual entitlement underlie male perpetration of NPSV [18]. Not only associated with strong attitudes on violence, male sexual entitlement is driven by hierarchical and marginalizing masculine norms, traditional gender roles, and notions of male superiority [19] that are best summed up in the concept of hypermasculinity [20]. Success and legitimacy within the male traditional gender role is accomplished by performing and projecting toughness, strength, and the ability to control women, which may be also achieved through sexual violence [18, 21]. Therefore, gender inequitable norms and hypermasculinity, as well as male sexual entitlement, are directly tied to NPSV perpetration (Fig. 1).

Moreover, the same inequitable gender norms and hypermasculinity that fuel male sexual entitlement are associated with several recognised risk factors for NPSV perpetration [12], thereby indirectly linking or mediating the relationship between the two (Fig. 1). Risky sexual behaviours, including early age at first intercourse [22, 23], engaging in transactional sex and having multiple concurrent sexual partners [18, 24, 25], have been found to be associated with rape perpetration in different contexts. Similarly, quantitative and qualitative studies have linked consumption of sexually explicit media, particularly violent pornography, to sexual violence [26–28].

There is widespread evidence of mental health- and substance misuse-related factors being drivers of different forms of NPSV perpetration too, including problem alcohol use [14, 29], drug use [25, 30], and poor mental health, for instance stress, post-traumatic stress disorder [31], and depression [32]. Relatedly, poor mental health and substance misuse are often described as being driven by harmful masculine norms. For instance, excessive alcohol use and binge drinking have been used to project “strong” masculine identities [33], and mental health issues including depression and suicidality can also be a by-product of traditional masculinity, which discourages help-seeking behaviours in men [12, 34]. Moreover, traditional masculine expectations of economic provision often clash with the reality of poverty and unemployment, making men feeling like they are not living up to the masculine ideal [35].

The direct and indirect links between gender inequitable norms and male NPSV perpetration have been shown extensively in studies conducted in high-income setting, particularly among US American college students [36, 37], and to a lesser extent in other contexts and populations. Studies focusing on young men in sub-Saharan Africa are mostly located in South Africa [21, 25] and occasionally in West African countries [28, 38], while little is known about perpetration of NPSV in the context of East Africa. With Tanzania being the largest country in the region, there is a need to further explore risk factors for male perpetration of NPSV in this setting to understand what drives acts of rape and sexual harassment, in order to indicate where and how future interventions to reduce NPSV incidence in the general population might work.

## Methods

### Sample and data collection

A cross-sectional survey was conducted among 1002 young men aged 18 to 24 between June 2021 and February 2022. Six wards were purposefully chosen in the Ilemela and Nyamagana districts of Mwanza, Tanzania, the second biggest city in the country. A random sample of 24 streets was selected across these wards, including densely-populated wards closer to the city centre and sparsely-populated wards on the outskirts of town. Once street boundaries were mapped, 120 points were randomly created on each map and tracked using GPS devices. Two households nearest to the point were chosen, and one young man per household was randomly selected to take part in the survey from a list of all eligible young men in that particular household. Trained fieldworkers administered a structured, multi-section questionnaire in Swahili, which had been previously back and forward translated from English and piloted extensively in the study context. The modules on violence perpetration were self-administered through Audio-Computer Assisted Self-Interviews to reduce underreporting [39]. Informed consent was obtained from all participants prior to data collection.

### Survey measures

The main outcome of this study was lifetime NPSV perpetration, which was defined as verbal sexual harassment, physical sexual harassment, and rape. Verbal sexual harassment (from now on “verbal harassment”) was measured with two questions: (1) ever making sexist jokes or offensive comments and (2) giving a woman unwanted sexual attention. Physical sexual harassment perpetration (“physical harassment”) was assessed through three questions: (1) ever touching a woman inappropriately against her will, (2) ever pressuring a woman into unwanted sexual activity and (3) ever sexually harassing a woman. Based on the 2021 systematic review by Ranganathan et al. [9], which found no existing validated measurement tools to assess sexual harassment specifically, we decided to formulate our own set of questions to capture the prevalence of sexual harassment perpetration, informed by qualitative work which had been conducted in the same setting. Five questions, similar to the ones used in the UN Multi-country Study on Men and Violence in Asia and the Pacific [14], were utilised to assess non-partner rape perpetration: (1) ever forcing a woman who was not your wife or girlfriend to have sex, (2) ever *trying to* have sex with a woman or girl when she was too drunk or drugged to consent, (3) ever having sex with a woman or girl when she was too drunk or drugged to consent, (4) together with other men, forcing a woman who is not wife/girlfriend to have sex, and (5) together with other men, having sex with a woman when she was too drunk or drugged to stop you (see Appendix 1). All questions had binary answer categories (yes, no) and answering “yes” to perpetrating one act was considered as perpetration of that specific form of violence.

### Risk factors

Potential risk factors for NPSV perpetration were drawn from the existing literature on both NPSV and IPV and grouped into sociodemographic, behaviour-related, and mental health-related variables. Socio-demographic risk factors were assessed such as age (continuous variable), education (no education, at least primary, at least secondary, college and university), employment status (yes, no), having ever been in a relationship (yes, no), and current relationship status (recategorized as married, one main partner, one or more casual partner(s), and no partner). Behaviour-related variables included age at sexual debut (never, 9–14, 15–19, 20–24), intercourse in the past 12 months (yes, no), having ever solicited transactional sex (yes, no, N/A), pornography consumption in the previous 12 months (never, sometimes, often), having multiple sexual partners (yes, no, N/A), having gambled in the past 12 months (yes, no). Mental health- and substance misuse-related risk factors collected were alcohol use (measured through the AUDIT score [40], categorised as abstainer [scored 0], low-risk consumption [1–7], harmful alcohol consumption [8–14], alcohol dependent [15 +]), drug use (yes, no), and depressive symptoms (measured through the PHQ-9 questionnaire [41], categorised as none/minimal [scored 1–4], mild [5–9], moderate to severe [10–27] depression).

### Statistical analysis

The data were analysed using STATA 17.0 taking into account potential clustering at the street level. Descriptive characteristics of our sample of 1002 men were computed (percentages for the three NPSV forms, as well as for each risk factor). Secondly, unadjusted logistic regression was used to estimate the strength of the association at the bivariate level between the three outcomes separately and each risk factor by calculating the corresponding odds ratios. Those risk factors that showed a strong effect (OR > 1.5) and were statistically significantly associated with each form of NPSV (*p* < 0.05) were then added to the respective multivariable logistic regression model. Additionally, dominance analysis was performed [42] to assess each variable’s effect on each from of NPSV perpetration and its effect in the presence of other variables, reflecting the indirect pathways shown in Fig. 1.

## Results

### Participant characteristics

Among the 1002 Tanzanian men in the sample, over two thirds were currently in paid employment (66.9%) and the majority had been in a relationship at some point in their life (82.6%). There was a fairly even spread of participants across ages 18–24 and across levels of educational attainment. Most men had either one current main partner (31.9%) or several casual partners (34.4%), with 24.6% having no partner at the moment and just under nine percent being married (Table 1).

**Table 1:** Sociodemographic, behavioural and health characteristics of sample (*N* = 1002) and bivariate associations with NPSV perpetration

		Verbal sexual harassment [*N* = 330 (32.93%)]			Physical sexual harassment [*N* = 188 (18.76%)]			Non-Partner rape [*N* = 86 (8.58%)]		
	N (%)	n / N (%)	Crude OR (95% CI)	*p*-value	n / N (%)	Crude OR (95% CI)	*p*-value	n / N (%)	Crude OR (95% CI)	*p*-value
**Socio-demographic risk factors**										
*Age*										
18	181 (18.06)	60/181 (33.15)			38/181 (20.99)			16/181 (8.84)		
19	168 (16.77)	58/168 (34.52)			36/168 (21.43)			9/168 (5.36)		
20	132 (13.17)	36/132 (27.27)			22/132 (16.67)			8/132 (6.06)		
21	173 (17.27)	58/173 (33.53)			28/173 (16.18)			15/173 (8.67)		
22	108 (10.78)	42/108 (38.89)			24/108 (22.22)			9/108 (8.33)		
23	126 (12.57)	42/126 (33.33)			21/126 (16.67)			14/126 (11.11)		
24	114 (11.38)	34/114 (29.82)	0.99 (0.93–1.06)	0.940	19/114 (16.67)	0.96 (0.88–1.03)	0.273	15/114 (13.16)	1.11 (0.99–1.25)	0.052
*Education*										
No education	108 (10.78)	36/108 (33.33)	1		23/108 (21.30)	1		15/108 (13.89)	1	
At least primary	383 (38.22)	117/383 (30.55)	0.88 (0.56–1.39)	0.581	69/383 (18.02)	0.81 (0.48–1.38)	0.441	35/383 (9.14)	0.62 (0.33–1.19)	0.152
At least secondary	407 (40.62)	141/407 (34.64)	1.06 (0.68–1.66)	0.799	77/407 (18.92)	0.86 (0.51–1.45)	0.579	28/407 (6.88)	0.46 (0.23–0.89)	0.022
College and university	104 (10.38)	36/104 (34.62)	1.06 (0.60–1.87)	0.844	19/104 (18.27)	0.83 (0.42–1.63)	0.581	8/104 (7.69)	0.52 (0.21–1.28)	0.152
*Employment*										
Yes	670 (66.87)	220/670 (32.84)	1		123/670 (18.76)	1		64/670 (9.55)	1	
No	332 (33.13)	110/332 (33.13)	1.01 (0.77–1.34)	0.925	65/332 (19.58)	1.08 (0.77–1.51)	0.642	22/332 (6.63)	0.67 (0.41–1.11)	0.122
*Ever had relationship*										
Yes	828 (82.63)	281/828 (33.94)	1		163/828 (19.69)	1		80/828 (9.66)	1	
No	174 (17.37)	49/174 (28.16)	0.76 (0.53–1.09)	0.141	25/174 (14.37)	0.68 (0.43–1.08)	0.104	6/174 (3.45)	0.33 (0.14–0.78)	0.011
*Current relationship status*										
Married	90 (8.98)	24/90 (26.67)	1		14/90 (15.56)	1		8/90 (8.89)	1	
One main partner	320 (31.94)	92/320 (28.75)	1.11 (0.65–1.88)	0.698	50/320 (15.62)	1.00 (0.53–1.92)	0.987	19/320 (5.94)	0.65 (0.27–1.53)	0.322
Casual partners	345 (34.43)	137/345 (39.71)	1.81 (1.08–3.03)	0.024	86/345 (24.93)	1.80 (0.97–3.35)	0.063	51/345 (14.78)	1.78 (0.81–3.90)	0.150
No partner	247 (24.65)	77/247 (31.17)	1.24 (0.73–2.13)	0.425	38/247 (15.38)	0.99 (0.51–1.92)	0.969	8/247 (3.24)	0.34 (0.12–0.94)	0.038
**Behavioural risk factors**										
* Age at first sex*										
Never	179 (17.86)	53/179 (29.61)	1		28/179 (15.64)	1		7/179 (3.91)	1	
9 to 14	93 (9.28)	26/93 (27.96)	0.92 (0.53–1.61)	0.776	20/93 (21.51)	1.48 (0.78–2.80)	0.231	8/93 (8.60)	2.31 (0.81–6.59)	0.117
15 to 19	654 (65.27)	231/654 (35.32)	1.30 (0.91–1.86)	0.154	127/654 (19.42)	1.30 (0.83–2.03)	0.251	64/654 (9.79)	2.66 (1.20–5.92)	0.016
20 to 24	76 (7.58)	20/76 (26.32)	0.85 (0.46–1.55)	0.595	13/76 (17.11)	1.11 (0.54–2.29)	0.771	7/76 (9.21)	2.49 (0.84–7.37)	0.099
* Sex in the past 12 m*										
No	619 (75.21)	59/204 (28.92)	1		41/204 (20.10)	1		15/204 (7.35)	1	
Yes	204 (24.79)	218/619 (35.22)	1.34 (0.95–1.88)	0.099	119/619 (19.22)	0.95 (0.64–1.41)	0.785	64/619 (10.34)	1.45 (0.81–2.61)	0.211
* Transactional sex ever*										
No	248 (24.75)	61/248 (24.60)	1		38/248 (15.32)	1		12/248 (4.84)	1	
Yes	575 (57.39)	216/575 (37.57)	1.84 (1.32–2.58)	< 0.0001	122/575 (21.22)	1.49 (0.99–2.22)	0.051	67/575 (11.65)	2.59 (1.38–4.89)	0.003
N/A	179 (17.86)	53/179 (29.61)	1.29 (0.84–1.98)	0.249	28/179 (15.64)	1.02 (0.60–1.74)	0.928	7/179 (3.91)	0.80 (0.31–2.07)	0.647
* Pornography consumption past 12 m*										
Never	543 (54.19)	140/543 (25.78)	1		76/543 (14.00)	1		39/543 (7.18)	1	
Sometimes	394 (39.32)	154/394 (39.09)	1.85 (1.40–2.44)	< 0.0001	89/394 (22.59)	1.79 (1.28–2.51)	0.001	36/394 (9.14)	1.30 (0.81–2.08)	0.277
Often	65 (6.49)	36/65 (55.38)	3.57 (2.11–6.04)	< 0.0001	23/65 (35.38)	3.36 (1.91–5.91)	< 0.0001	11/65 (16.92)	2.63 (1.27–5.44)	0.009
* Multiple sexual partners*										
No	462 (46.11)	115/462 (24.89)	1		71/462 (15.37)	1		31/462 (6.71)	1	
Yes	299 (29.84)	139/299 (46.49)	2.62 (1.92–3.57)	< 0.0001	78/299 (26.09)	1.94 (1.35–2.79)	< 0.0001	47/299 (15.72)	2.59 (1.60–4.19)	< 0.0001
N/A	241 (24.05)	76/241 (31.54)	1.39 (0.98–1.96)	0.061	39/241 (16.18)	1.06 (0.69–1.63)	0.778	8/241 (3.32)	0.48 (0.21–1.05)	0.068
* Gambling past 12 m*										
No	790 (78.84)	233/790 (29.49)	1		125/790 (15.82)	1		51/790 (6.46)	1	
Yes	212 (21.16)	97/212 (45.75)	2.02 (1.48–2.75)	< 0.0001	63/212 (29.72)	2.25 (1.58–3.19)	< 0.0001	35/212 (16.51)	2.86 (1.80–4.54)	< 0.0001
**Mental health-related risk factors**										
* Alcohol use*										
None	806 (80.44)	242/806 (30.02)	1		137/806 (17.00)	1		55/806 (6.82)	1	
Low risk	103 (10.28)	43/103 (41.75)	1.67 (1.10–2.54)	0.017	21/103 (20.39)	1.25 (0.75–2.09)	0.393	6/103 (5.83)	0.84 (0.35–2.01)	0.703
Harmful	66 (6.59)	30/66 (45.45)	1.94 (1.17–3.22)	0.010	17/66 (25.76)	1.69 (0.95–3.03)	0.076	15/66 (22.73)	4.02 (2.12–7.60)	< 0.0001
Alcohol dependent	27 (2.69)	15/27 (55.56)	2.91 (1.34–6.32)	0.007	13/27 (48.15)	4.53 (2.08–9.86)	< 0.0001	10/27 (37.04)	8.03 (3.51–18.38)	< 0.0001
* Drug use*										
No	967 (96.51)	310/967 (32.06)	1		176/967 (18.20)	1		75/967 (7.76)	1	
Yes	35 (3.49)	20/35 (57.14)	2.82 (1.43–5.59)	0.003	12/35 (34.29)	2.34 (1.14–4.80)	0.020	11/35 (31.43)	5.45 (2.57–11.56)	< 0.0001
* Depressive symptoms*										
None	618 (61.68)	164/618 (26.54)	1		87/618 (14.08)	1		35/618 (5.66)	1	
Mild	301 (30.04)	132/301 (43.85)	2.16 (1.62–2.89)	< 0.0001	77/301 (25.58)	2.10 (1.49–2.96)	< 0.0001	39/301 (12.96)	2.48 (1.53–4.00)	< 0.0001
Moderate to severe	83 (8.28)	34/83 (40.96)	1.92 (1.20–3.08)	0.007	24/83 (28.92)	2.48 (1.47–4.20)	0.001	12/83 (14.46)	2.81 (1.40–5.67)	0.004

Most young men reported having their first sexual encounter between the ages of 15 and 19 (65.3%), and smaller proportions between the ages of nine and 14 (9.3%) and 20 to 24 (7.6%), with 17.9% stating that they never had sexual intercourse. Of those who already had engaged in sexual activities, about three quarters also had sex in the previous twelve months (75.2%) and over half had ever solicited transactional sex (57.4%). Just over half of the sample reported never having consumed sexually explicit media in the past year (54.2%), whereas 39.3% reported consuming pornographic materials “sometimes” and 17.9% “often”. Under half of the young men surveyed had just one sexual partner (46.1%) and 29.8% admitted to having multiple sexual partners besides their most current or recent main partner. Finally, 21.2% of our sample reported having gambled money over the past twelve months (Table 1).

Most respondents said they did not consume alcohol in the previous year (80.4%), 10.3% were considered “low risk” consumers, 6.6% displayed harmful levels of alcohol consumption, and 2.7% were classified as “alcohol dependant”. The majority reported not using drugs, with 3.5% admitting to smoking cannabis. Finally, 30.0% of the respondents report mild depressive symptoms and 8.3% have moderate to severe depression (Table 1).

Out of the 1002 young men that made up our sample, 32.9% reported having ever perpetrated verbal harassment (*n* = 330) and 18.8% physical harassment (*n* = 118). Additionally, 8.6% of the men reported committing rape (*n* = 86) (Table 1, top row), with 1.4% reporting involvement in multi-perpetrator rape.

### Crude and adjusted associations of potential risk factors with NPSV

#### Verbal harassment

The only socio-demographic risk factor associated with verbal harassment was current relationship status: having one or more casual partners was significantly associated with higher odds of perpetration (OR = 1.81, 95% CI = 1.08—3.03) compared to being married, having a single partner or no partner. As for behavioural risk factors, having ever engaged in transactional sex (OR = 1.84, 95% CI = 1.32—2.58), having consumed pornography in the previous twelve months (*sometimes*: OR = 1.85, 95% CI = 1.40—2.44; *often*: OR = 3.57, 95% CI = 2.11—6.04), having multiple concurrent sexual partners (OR = 2.62, 95% CI = 1.92—3.57) compared to none, and having gambled in the past year (OR = 2.02, 95% CI = 1.48—2.75) were all significantly associated with perpetration of verbal harassment. All three mental health-related risk factors were associated with verbal harassment perpetration in the bivariate analysis: alcohol use (*low risk*: OR = 1.67, 95% CI = 1.10—2.54; *harmful*: OR = 1.94, 95% CI = 1.17—3.22; *alcohol dependent*: OR = 2.91, 95% CI = 1.34—6.32), drug use (OR = 2.82, 95% CI = 1.43—5.59) and depressive symptoms (*mild*: OR = 2.16, 95% CI = 1.62—2.89; *moderate to severe*: OR = 1.92, 95% CI = 1.20—3.08) (Table 1).

After adjusting for co-variates, in the final model for verbal harassment, pornography consumption (*sometimes*: aOR = 1.47, 95% CI = 1.02—2.14; *often*: aOR = 2.42, 95% CI = 1.20—4.86), multiple sexual partners (aOR = 2.01, 95% CI = 1.50—2.69), mild depressive symptoms (aOR = 1.96, 95% CI = 1.48—2.60), gambling (aOR = 1.46, 95% CI = 1.03—2.07) and transactional sex (aOR = 1.60, 95% CI = 1.04—2.46) remained significant predictors of verbal harassment perpetration (Table 2).

**Table 2:** Adjusted associations between risk factors and NPSV perpetration

Verbal sexual harassment			Physical sexual harassment			Non-partner rape		
Risk factor	Adjusted OR (95% CI)*	*p*-value	Risk factor	Adjusted OR (95% CI)†	*p*-value	Risk factor	Adjusted OR (95% CI)‡	*p-*value
*Pornography consumption past 12 m*			*Alcohol use*			*Drug use*		
Never	1		None	1		No	1	
Sometimes	1.47 (1.02–2.14)	0.040	Low risk	1.02 (0.55–1.88)	0.953	Yes	4.88 (1.80–13.18)	0.002
Often	2.42 (1.20–4.86)	0.013	Harmful	1.12 (0.56–2.24)	0.740	*Gambling past 12 m*		
*Alcohol use*			Alcohol dependent	2.55 (0.99–6.56)	0.052	No	1	
None	1		*Pornography consumption past 12 m*			Yes	2.56 (1.53–4.27)	< 0.0001
Low risk	1.36 (0.93–2.00)	0.110	Never	1		*Depressive symptoms*		
Harmful	1.22 (0.70–2.13)	0.489	Sometimes	1.44 (1.06–1.96)	0.020	None	1	
Alcohol dependent	1.53 (0.64–3.67)	0.337	Often	2.15 (1.26–3.66)	0.005	Mild	2.19 (1.42–4.39)	< 0.0001
*Multiple sexual partners*			*Depressive symptoms*			Moderate to severe	2.16 (1.02–4.58)	0.043
No	1		None	1				
Yes	2.01 (1.50–2.69)	< 0.0001	Mild	1.87 (1.40–2.50)	< 0.0001			
N/A	1.99 (1.16–3.42)	0.012	Moderate to severe	1.87 (0.99–3.53)	0.055			
*Depressive symptoms*			*Gambling past 12 m*					
None	1		No	1				
Mild	1.96 (1.48–2.60)	< 0.0001	Yes	1.66 (1.19–2.33)	0.003			
Moderate to severe	1.49 (0.95–2.34)	0.082	*Multiple sexual partners*					
*Gambling past 12 m*			No	1				
No	1		Yes	1.52 (1.07–2.17)	0.020			
Yes	1.46 (1.03–2.07)	0.032	N/A	1.09 (0.64–1.86)	0.747			
*Transactional sex ever*								
No	1							
Yes	1.60 (1.04–2.46)	0.031						
N/A	1.02 (0.64–1.62)	0.935						

^*^Adjusted for age, pornography consumption, alcohol use, multiple sexual partners, depression, gambling, transactional sex

†Adjusted for age, alcohol use, pornography consumption, depression, gambling, multiple sexual partners

‡Adjusted for age, drug use, gambling, depression

#### Physical harassment

No socio-demographic variables were associated with perpetration of physical harassment. Three behavioural risk factors were associated with the outcome in the bivariate analysis: pornography consumption (*sometimes*: OR = 1.79, 95% CI = 1.28—2.51; *often*: OR = 3.36, 95% CI = 1.91—5.91), having multiple sexual partners (OR = 1.94, 95% CI = 1.35—2.79) compared to none, and gambling (OR = 2.25, 95% CI = 1.58—3.19). All three mental health-related risk factors were significantly associated with this type of NPSV: alcohol use, but only in the *alcohol dependent* category (OR = 4.53, 95% CI = 2.08—9.86), drug use (OR = 2.34, 95% CI = 1.14—4.80) and depressive symptoms (*mild*: OR = 2.10, 95% CI = 1.49—2.96; *moderate to severe*: OR = 2.48, 95% CI = 1.47—4.20) (Table 1).

In the adjusted model, physical sexual harassment remained associated with pornography consumption (*sometimes*: aOR = 1.44, 95% CI = 1.06—1.96; *often*: aOR = 2.15, 95% CI = 1.26—3.66), mild depressive symptoms (aOR = 1.87, 95% CI = 1.40—2.50), gambling (aOR = 1.66, 95% CI = 1.19—2.33) and multiple sexual partners (aOR = 1.52, 95% CI = 1.07—2.17) (Table 2).

#### Non-partner rape

Three socio-demographic variables were found to be associated with lower odds of rape perpetration: having achieved at least secondary education (OR = 0.46, 95% CI = 0.23—0.89), as well as never having had a relationship (OR = 0.33, 95% CI = 0.14—0.78) and currently having no partner (OR = 0.34, 95% CI = 0.12—0.94). Several behaviour-related risk factors were associated with higher odds of committing rape in the bivariate models: age at first sex between 15 and 19 years (OR = 2.66, 95% CI = 1.20—5.92), soliciting transactional sex (OR = 2.59, 95% CI = 1.38—4.89), frequent pornography consumption (OR = 2.63, 95% CI = 1.27—5.44), multiple sexual partners (OR = 2.59, 95% CI = 1.60—4.19), and gambling (OR = 2.86, 95% CI = 1.80—4.54). A significant association of rape emerged with all three mental health-related risk factors: alcohol use (*harmful*: OR = 4.02, 95% CI = 2.12—7.60; *alcohol dependent*: OR = 8.03, 95% CI = 3.51—18.38), drug use (OR = 5.45, 95% CI = 2.57—11.56) and depressive symptoms (*mild*: OR = 2.48, 95% CI = 1.53—4.00; *moderate to severe*: OR = 2.81, 95% CI = 1.40—5.67) (Table 1).

In the adjusted models, perpetration of non-partner rape was associated with drug use (aOR = 4.88, 95% CI = 1.80—13.18), gambling (aOR = 2.56, 95% CI = 1.53—4.27), and depressive symptoms (*mild*: OR = 2.19, 95% CI = 1.42—4.39; *moderate to severe*: OR = 2.16, 95% CI = 1.02—4.58) (Table 2).

### Dominance analysis

Dominance analysis was conducted to understand the order of strength with which each risk factor was associated with the three different outcomes. The three strongest risk factors associated with verbal harassment were pornography consumption, depressive symptoms and gambling. Similarly, the three strongest risk factors for physical harassment were pornography consumption, depressive symptoms and gambling. For rape perpetration, the three strongest risk factors are drug use, gambling and depressive symptoms. Dominance statistics are presented in Appendix 2.

## Discussion

This study sought to capture the prevalence of perpetration of three different forms of NPSV and to understand which risk factors are associated with each form of NPSV in our sample of young Tanzanian men. We explored several known risk factors from the existing literature on both IPV and NPSV perpetration, with our data showing strong and consistent associations with a number of them, especially in the behavioural and mental health and substance misuse categories.

Data from the young Tanzanian men in our study showed that rape perpetration is worryingly high, with nearly one in ten participants admitting to forcing a woman or girl to have sex against her will or when she was incapable of consenting, a figure higher than national estimate provided by women (3.3%) and regional estimate for Mwanza (6.8%) of non-partner rape experience [7]. This finding is consistent with the even higher rates of verbal and physical sexual harassment we uncovered in our sample (32.9% and 18.8%, respectively).

Our results confirm that many of the factors described in previous studies as potential drivers of perpetration of NPSV, namely early age of sexual debut, engaging in transactional sex, consuming sexually explicit media and having multiple concurrent sexual partners [23], were all associated with non-partner rape perpetration in our sample, and the latter two were also associated with verbal and physical harassment. These risk factors can all be considered expressions of hypermasculinity and conformity with dominant gender norms that prescribe sexual prowess and domination in men, as well as male sexual entitlement. Evidence from different contexts shows that endorsement of inequitable gender norms is also associated with increased odds of IPV perpetration [43, 44].

Gambling was also found to be associated with NPSV, and in previous research has been shown to be associated with IPV perpetration as well [45, 46]. The association we discovered with NPSV could be understood as another behaviour reflecting the hypermasculine ideal, with risk-taking being a core component of traditional masculine norms. At the same time, it may be that these behaviours are indicative of poor impulse control being a common denominator for the two, as gambling has been documented to be positively correlated with impulsivity [47], although the evidence on the role of impulsivity on sexual violence perpetration so far has been mixed [23].

Confirming what we gathered from the literature on both IPV and NPSV, the same mental health-related risk factors that predict perpetration of the former, such as problem alcohol use [48], drug use [49], and depressive symptoms [50], were strongly associated with all three forms of NPSV perpetration in our analysis. Interestingly, alcohol use lost significance in the adjusted models, despite being a major risk factor for NPSV perpetration in other settings [51]. At the same time, it should be noted that alcohol consumption in this particular sample is comparatively low, with 80.4% reporting abstaining from drinking. Furthermore, in the adjusted models we see that mild depressive symptoms remain associated with all three forms of NPSV perpetration, whereas the association with moderate to severe depressive symptoms loses some of its strength. This could point to some other factors concurrent with severe depression better predicting the occurrence of NPSV perpetration, or that many young men who suffer from severe mental health issues are not in a place to perpetrate this violence. One could also hypothesise that some men suffering from mild depressive symptoms perpetrate NPSV to exercise control over women as a way to improve their self-esteem [52], but more evidence is needed to ground this finding.

**Fig. 2:**
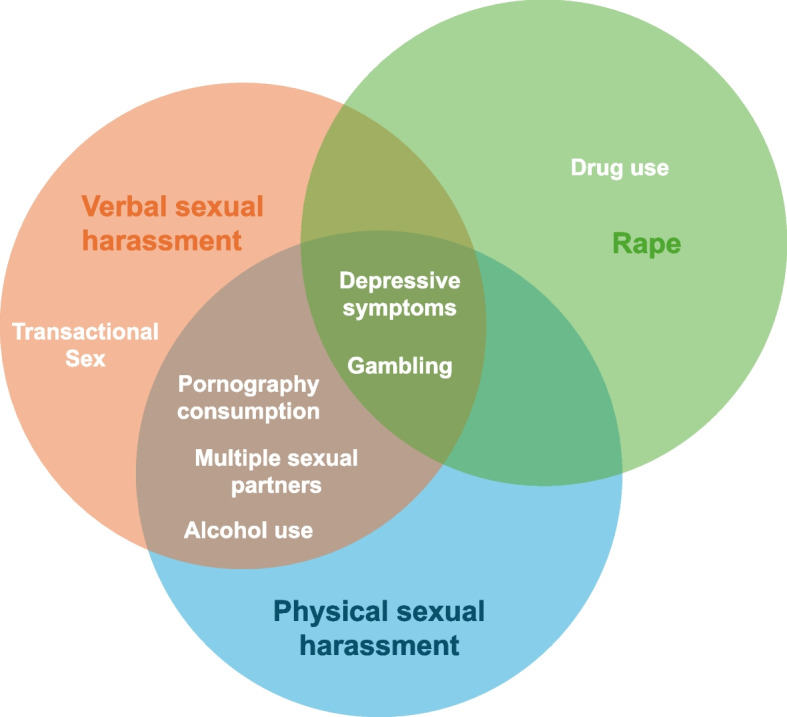
Overlaps between risk factors associated with verbal and physical sexual harassment and non-partner rape

As understanding risk factors behind perpetration is crucial to be able to reduce NPSV, we were able to delineate significant and interesting overlaps between predictors of verbal harassment, physical harassment and rape (Fig. 2). Exposure to sexually explicit media in particular came out as the strongest predictors for both forms of sexual harassment in the dominance analysis, something that has also been established for IPV perpetration [53], with higher odds of perpetration of verbal harassment than physical harassment. This finding is corroborated by evidence from a meta-analysis of studies on pornography consumption, which found stronger associations with verbal sexual aggression than physical aggression [54], suggesting that it is important to differentiate between different types of sexually aggressive behaviour to better understand the influence of sexually explicit media. Moreover, we saw a stronger association of either form of sexual harassment with more frequent pornography consumption, whereas most studies display inconsistent findings between frequency of consumption and sexual aggression [55, 56].

We found a particularly strong and persistent association of mild depressive symptoms with perpetration of rape, verbal and physical harassment in the final models, underscoring how poor mental health is a risk factor for perpetration of NPSV. There is evidence from pooled data from LMICs that depression is associated with male NPSV perpetration [15], but the mechanisms behind this are still poorly understood. A possible explanation could be that depression stems from a sense of unfulfillment regarding traditional masculine norms [12]. While we did not find a direct association between employment status and NPSV perpetration, we know that young people being unemployed and unable to provide a stable livelihood are considered a significant challenge in this setting, and despite two thirds of our sample reporting being currently in employment, there is evidence that young men are pressured to take up physically demanding, poorly paying, unpleasant income-generating activities in order to have some income [57], potentially impacting their mental health. Moreover, the men in our sample are relatively young (aged 18-24) and some of them might still be in school, and therefore not employed. So whereas we cannot consider lack of employment a risk factor directly impacting the perpetration of NPSV, in this context, using violence may be an attempt to reaffirm one’s masculinity to contrast this sense of “failure” in the face of an unattainable masculine ideal. Moreover, as most of the current literature explores depression and post-traumatic symptoms in those *experiencing* sexual violence, but not in perpetrators, we recommend that future research is conducted on male mental health to better understand its role in NPSV perpetration.

The consistent associations found between non-partner rape, verbal and physical harassment perpetration and a number of risky sexual behaviours reinforces the idea that these may cluster together and be indicative of inequitable gender norms and concepts of hypermasculinity based on domination, promiscuity and male sexual entitlement. Similarly, having found significant associations with risk factors like substance abuse, gambling and depression underscores the need to question traditional masculine gender norms, the pressure they place on young men, and whether NPSV perpetration is seen as an avenue to reaffirm one’s masculinity when young men otherwise fail to live up to these hypermasculine ideals.

### Strengths and limitations

This analysis has contributed to the existing body of knowledge on risk factors for NPSV in several ways. It included sexual harassment as an outcome, a phenomenon where evidence remains lacking, and it analysed verbal and physical sexual harassment separately, showing that they have similar but not entirely overlapping risk factors. Moreover, after confirming patterns of association with already researched risk factors, it showed associations with some previously unexplored ones, such as gambling.

Our analysis presents some limitations: first, data were collected cross-sectionally at a single time point, therefore we cannot infer any causation or establish temporality between the risk factors and our three NPSV outcomes, only associations. Second, we measured sensitive behaviours, hence our estimates might be underreported because of social desirability bias, something we tried to reduce by collecting data on violence perpetration through self-completion of these questions. Third, some of the risk factors (drug consumption and moderate to severe depression, for example) and outcomes (rape perpetration) were not frequently reported, therefore some of the associations we found may be spurious and should be interpreted with a degree of caution. Fourth, in our questionnaire we only measured socio-demographic and behavioural risk factors, which we assumed to be proxies for inequitable gender norms, masculinity, and attitudes of male sexual entitlement. We could not establish the direct pathways from these to NPSV perpetration, only showcase how the analysed risk factors, which might well be motivated by hypermasculinity and male sexual entitlement, are associated with rape, verbal and physical harassment in our sample. Future studies should seek to capture gender norms and male sexual entitlement directly and investigate how those are related to different forms of NPSV perpetration. While we acknowledge that men may experience sexual harassment and/or non-partner rape too, in this study we decided to focus on male-to-female perpetration of NPSV given the comparatively higher prevalence rates of female experience of violence. Moreover, our study only takes in-person violence into account and does not differentiate between this and online/technology-facilitated harassment and sexual violence, both phenomena which are on the rise across the world. A study measuring technology-facilitated gender-based violence is currently being conducted in this same geographic context among young men and women, and more evidence needs to be gathered in the future on this and other forms of GBV. Finally, despite confirming our findings with the current literature, the results of our analysis are not entirely generalisable outside of the population of young Tanzanian men who took part in our study.

## Conclusion and recommendations

This study revealed a high prevalence of rape and sexual harassment perpetration among young men living in Mwanza, Tanzania. The analysis found a range of risk factors consistently associated with all forms of NPSV, namely pornography consumption, multiple sexual partners, depressive symptoms and gambling, which can be thought of as indicators for traditional masculine gender norms that exalt sexual prowess, promiscuity, and risky health behaviours. Depression, substance abuse, and gambling can also be understood within the matrix of behaviours engendered by traditional masculinity, making men more risk-prone and isolated, as well as more likely to assault women.

Whilst policies should be put in place to elicit a substantial response from criminal justice systems to effectively prosecute rape perpetration and criminalise sexual harassment, the overlaps we found between risk factors for different forms of NPSV shows us a significant potential for joint action at the programmatic level. As our study indicates that several behaviours are best understood as a manifestation of harmful gender norms associated with NPSV perpetration, the design and evaluation of interventions addressing hypermasculinity and sexual entitlement will be key in reducing future incidence of NPSV perpetration. The involvement of men in shaping future programmatic efforts – questioning the motives behind these behaviours, the need to use violence to reaffirm one’s gender role, as well as promoting alternative forms of masculinity that will benefit men in the long term – is vital for the success of these interventions.

Concurrent with ongoing research on IPV, more research needs to occur on forms of violence against women which are less commonly studied, such as sexual harassment. Common working definitions and measurement tools need to be crafted to establish reliable estimates nationally and globally, and studies need to be conducted to deepen understanding of prevalence rates, potential causes, and key determinants of perpetration. The association of some of the risk factors we uncovered, namely relationship status and gambling, with NPSV perpetration should be investigated in different geographic settings, to confirm the role they play in this phenomenon. A number of other potential risk factors, such as various forms of emotional dysregulation and aggression, could also be further explored to expand our knowledge-base on NPSV.

We see a pressing need to consider these factors jointly and how they all fit within a broader framework of harmful masculine gender norms that takes into account the harms done both to women and girls, but also to men themselves.

## Supplementary Information

Supplementary Material 1. Appendix 1, Survey questions on NPSV perpetration, table.

Supplementary Material 2. Appendix 2, Dominance analysis results for risk factors associated with NPSV perpetration, table.

## Data Availability

The datasets generated during and/or analyzed during the current study are not publicly available due to the sensitive nature of some of the topics collected, but are available from the corresponding author on reasonable request.

## Abbreviations

AUDIT: Alcohol Use Disorders Identification Test
IPV: Intimate Partner Violence
LMIC: Low- and Middle-Income Country
NPSV: Non-Partner Sexual Violence
PHQ-9: Patient Health Questionnaire-9
