# Correction: Understanding non‑partner sexual violence perpetration in young Tanzanian men: a cross‑sectional study

**DOI:** 10.1186/s12889-025-23782-1

**Published:** 2025-07-17

**Authors:** Rebecca Brambilla, Gerry Mshana, Neema Mosha, Andrew Gibbs, Donati Malibwa, Saidi Kapiga, Heidi Stöckl

**Affiliations:** 1 Institute for Medical Information Processing, Biometry and Epidemiology (IBE), Faculty of Medicine, LMU Munich, Munich, Germany; 2 Pettenkofer School of Public Health, Munich, Germany; 3 Mwanza Intervention Trials Unit (MITU), Mwanza, Tanzania; 4 Department of Psychology, University of Exeter, Exeter, UK; 5 Gender and Health Research Unit, South African Medical Research Council, Cape Town, South Africa; 6 Department of Global Health and Development, London School of Hygiene and Tropical Medicine (LSHTM), London, UK

**Correction: BMC Public Health 25**,** 2000 (2025)**

**https://doi.org/10.1186/s12889-025-23248-4**

Following publication of the original article [1], the authors reported an error found in Tables 1 and 2. In Table 1, the heading “Socio-demographic risk factors” is not part of the table heading and should be moved under the table heading as a sub-heading. In Table 2, the following notes were captured as footnotes.

*Adjusted for age, pornography consumption, alcohol use, multiple sexual partners, depression, gambling, transactional sex

†Adjusted for age, alcohol use, pornography consumption, depression, gambling, multiple sexual partners

‡Adjusted for age, drug use, gambling, depression

The original article [1] has been updated.
