# Supplementary material for: Understanding non-partner sexual violence perpetration in young Tanzanian men: a cross-sectional study
Source: BMC Public Health. 2025 May 30;25:2000. doi: 10.1186/s12889-025-23248-4 (PMC12123990; doi:10.1186/s12889-025-23248-4)
Supplement: Supplementary file 1 — Supplementary Material 1. Appendix 1, Survey questions on NPSV perpetration, table. [file 12889_2025_23248_MOESM1_ESM.docx]

# Appendix 1

Survey questions on NPSV perpetration

| *Questions* | *Answers* |
| --- | --- |
| Verbal sexual harassment |  |
| Have you ever made sexist jokes or offensive comments to a woman at work, school or in public? | Yes  No |
| Have you ever given a woman at work, school or in public unwanted sexual attention, for example by asking her out even though she said no? | Yes  No |
| Physical sexual harassment |  |
| Have you ever touched a woman at work, school or in public inappropriately against their will? | Yes  No |
| Have you ever pressured a woman at work, school or in public into unwanted sexual activity, for example by trying to bribe or threaten her to have sex with you? | Yes  No |
| Have you ever sexually harassed a woman? | Yes  No |
| Non-partner rape |  |
| Have you ever forced a woman who was not your wife or girlfriend to have sex with you? | Yes  No |
| Have you ever tried to have sex with a woman or girl when she was too drunk or drugged to say whether she wanted it or not? | Yes  No |
| Have you ever had sex with a woman or girl when she was too drunk or drugged to say whether she wanted it or not? | Yes  No |
| Have you and other men ever had sex with a woman at the same time when she didn’t consent to sex or you forced her? | Yes  No |
| Have you and other men ever had sex with a woman at the same time when she was too drunk or drugged to stop you? | Yes  No |
