# Supplementary material for: Understanding non-partner sexual violence perpetration in young Tanzanian men: a cross-sectional study
Source: BMC Public Health. 2025 May 30;25:2000. doi: 10.1186/s12889-025-23248-4 (PMC12123990; doi:10.1186/s12889-025-23248-4)
Supplement: Supplementary file 2 — Supplementary Material 2. Appendix 2, Dominance analysis results for risk factors associated with NPSV perpetration, table. [file 12889_2025_23248_MOESM2_ESM.docx]

# Appendix 2

Dominance analysis results for risk factors associated with NPSV perpetration

| **Verbal Sexual Harassment** | | | |
| --- | --- | --- | --- |
| **Ranking** | **Risk factor** | **Dominance Stat** | **Standaridsed D S** |
| 1 | *Pornography consumption past 12m* | 0.0250 | 0.3198 |
| 2 | *Depressive symptoms* | 0.0169 | 0.2164 |
| 3 | *Gambling past 12m* | 0.0116 | 0.1483 |
| 4 | *Multiple sexual partners* | 0.0116 | 0.1480 |
| 5 | *Alcohol use* | 0.0101 | 0.1288 |
| 6 | *Transactional sex ever* | 0.0029 | 0.0372 |
| 7 | Age | 0.0001 | 0.0015 |
|  |  |  |  |
| **Physical Sexual Harassment** | | | |
| **Ranking** | **Risk factor** | **Dominance Stat** | **Standaridsed D S** |
| 1 | *Pornography consumption past 12m* | 0.0164 | 0.2824 |
| 2 | *Depressive symptoms* | 0.0160 | 0.2764 |
| 3 | *Gambling past 12m* | 0.0135 | 0.2325 |
| 4 | *Alcohol use* | 0.0097 | 0.1677 |
| 5 | Age | 0.0014 | 0.0245 |
| 6 | *Multiple sexual partners* | 0.0010 | 0.0165 |
|  |  |  |  |
| **Non-partner rape** | | | |
| **Ranking** | **Variable** | **Dominance Stat** | **Standaridsed D S** |
| 1 | *Drug use* | 0.0224 | 0.3989 |
| 2 | *Gambling past 12m* | 0.0182 | 0.3227 |
| 3 | *Depressive symptoms* | 0.0122 | 0.2173 |
| 4 | Age | 0.0034 | 0.0610 |
|  |  |  |  |
